# Body mass index and dyslipidemia influence responses to anti-PD-1 immunotherapy in colorectal cancer

**DOI:** 10.1080/07853890.2026.2624219

**Published:** 2026-08-24

**Authors:** Shi-Hong Chen, Le-En Liao, Zhen-Lin Hou, Kai Han, Wu Jiang, Pei-Rong Ding, Chen-Zhi Zhang

**Affiliations:** Departments of Colorectal Surgery, State Key Laboratory of Oncology in South China, Collaborative Innovation Center for Cancer Medicine, Sun Yat-sen University Cancer Center, Guangzhou, Guangdong Province, China

**Keywords:** Body mass index, dyslipidemia, anti-PD-1 outcome, colorectal cancer

## Abstract

**Background:**

Obesity is one of the primary risk factors for colorectal cancer (CRC) development, but  the impact of obesity on therapeutic responses to anti-programmed cell death protein 1 (PD-1) immunotherapy in CRC remains unclear. This retrospective study investigates relationships between body mass index (BMI), dyslipidemia, and clinical characteristics in immune checkpoint inhibitors (ICIs)-treated CRC patients, while analyzing associated metabolic variations.

**Methods:**

Progression-free survival (PFS) was assessed in CRC patients receiving anti-PD-1 therapy, categorized by BMI and dyslipidemia status. Comprehensive lipidomic profiling was performed using ultra-performance liquid chromatography-tandem mass spectrometry (UPLC-MS/MS) to identify metabolic mechanisms underlying therapeutic responses.

**Results:**

This study enrolled 142 CRC patients who completed ≥ 2 cycles of anti-PD-1 therapy. Patients with BMI ≥ 24 kg/m^2^ (overweight per Chinese criteria) showed significantly longer PFS compared to normal BMI counterparts (*p* = 0.001). Multivariate analysis confirmed BMI as an independent prognostic factor for PFS. Moreover, patients with dyslipidemia exhibited extended PFS. Additionally, lipidomic analysis revealed differential pre-treatment levels of phosphatidylcholine (PC), phosphatidylethanolamine-ether (PE-O) and carnitine between the recurrence (R) and non-recurrence (NR) groups, accompanied by an enrichment trend in glycerophospholipid metabolism.

**Conclusion:**

Our findings show that obesity correlates with enhanced ICIs efficacy in CRC patients, potentially mediated through glycerophospholipid metabolic reprogramming. Furthermore, this association is more pronounced in the janus kinases 1/2 (JAK1/2) and β2-microglobulin (B2M) wild-type subgroup. These results support the potential utility of BMI as a predictive biomarker and highlight the need for further exploration of lipid-modulating combination therapeutic strategies.

## Introduction

Colorectal cancer (CRC) is the third most common malignancy and ranks as the third leading cause of cancer-related death worldwide, underscoring the urgent need for enhanced therapeutic approaches [1]. Immune checkpoint inhibitors (ICIs), particularly anti-programmed cell death protein 1 (PD-1) agents, have revolutionized cancer treatment. However, their effectiveness in CRC is largely restricted to the microsatellite instability-high (MSI-H) subtype, which represents approximately 5% of metastatic CRC cases [2,3]. Concerningly, even within this limited population, primary resistance occurs in 40%-70% of patients, with 10%-28% exhibiting early progression [4,5]. Given these challenges, there is an urgent need to identify reliable and accessible biomarkers predictive of prognosis in CRC patients undergoing anti-PD-1 therapy, to guide patient stratification and optimize treatment decisions.

Body mass index (BMI) is a commonly used indicator of body fat mass [6]. Numerous studies have demonstrated that obesity is significantly associated with an increased risk of cancer [7–9]. Paradoxically, emerging evidence indicates that obesity may predict improved responses to immunotherapy, a phenomenon called the ‘obesity paradox’ [10–14]. Accumulating studies have demonstrated a positive association between high BMI and improved outcomes with ICIs in metastatic renal cell carcinoma and non-small cell lung cancer [15–17]. However, the prognostic value of BMI in CRC patients receiving ICIs remains unclear. Moreover, the mechanisms underlying this association – particularly the interplay between host metabolic status and tumor metabolism – are not fully understood.

 In this study, we aim to investigate the prognostic value of BMI in CRC patients treated with ICIs. Additionally, based on the pathophysiological linkage between BMI and dyslipidemia, we examined whether lipidomic reprogramming mechanistically connects BMI status with differential anti-PD-1 therapeutic outcomes. Lipidomic profiling was performed to delineate metabolic pathway divergences between CRC patients in the recurrence (R) and non-recurrence (NR) groups, with particular emphasis on crosstalk between host lipid metabolism and antitumor immunity.

## Methods

### Patient characteristics

A total of 486 patients diagnosed with CRC and treated with ICIs between January 1, 2019, and December 31, 2022, were extracted from the ‘Big Data Intelligence Framework’ at the Sun Yat-sen University Cancer Center. All patients received at least two doses of PD-1 inhibitor therapy, including Camrelizumab, Nivolumab, Pembrolizumab, Sintilimab, Tislelizumab, or Toripalimab. Exclusion criteria were as follows: enrollment in ongoing clinical trials (*n* = 205), missing BMI data (*n* = 76), loss to follow-up (*n* = 39), incomplete PD-1 inhibitor therapy (*n* = 28), or discontinuation due to severe immune-related adverse events (*n* = 22). Following these exclusions, 142 patients were included in the final analysis.

Baseline demographic and clinicopathological characteristics were obtained through manual chart review and used in subsequent statistical analysis. Data collected included age, sex, height, weight, histological subtype, primary tumor location, TNM stage, Eastern Cooperative Oncology Group (ECOG) performance status, microsatellite instability (MSI) and Janus kinases 1/2 (JAK1/2) or β2-microglobulin (B2M) status.

### Baseline BMI and lipid profile assessment

BMI assessments were conducted within 30 days prior to treatment initiation and calculated as weight in kilograms divided by height in meters squared (kg/m^2^). Categorization was performed at baseline using the World Health Organization (WHO) cutoff points adapted for the Chinese population [18]. Comorbidities were identified based on medical records, and only metabolic-related diseases were categorized as comorbidities for this study. Accordingly, patients were classified into two subgroups: normal weight (BMI < 24 kg/m^2^) and overweight (BMI ≥ 24 kg/m^2^). Dyslipidemia was defined as meeting ≥ 1 of the following criteria: total cholesterol (TC) > 5.69 mmol/L, low-density lipoprotein cholesterol (LDL-C) > 3.1 mmol/L, triglycerides (TG) > 1.7 mmol/L, or high-density lipoprotein cholesterol (HDL-C) < 1.16 mmol/L.

### Outcomes definitions and assessment

The primary outcome of this study was progression-free survival (PFS), defined as the interval between anti-PD-1 initiation and disease progression confirmed by investigator assessment or death from any cause. Secondary outcomes comprised overall survival (OS) and incidence of immune-related adverse events (irAEs). OS was defined as the interval from the initiation of anti-PD-1 therapy to death from any cause. Median follow-up was estimated using the reverse Kaplan-Meier method. Therapeutic outcomes and tumor response were assessed according to Response Evaluation Criteria in Solid Tumors (RECIST) version 1.1 criteria [19], including complete response (CR), partial response (PR), stable disease (SD), and progressive disease (PD). During follow-up, patients with disease recurrence (local/distant) were assigned to the R (Recurrence) group, while those without recurrence constituted the NR (Non-Recurrence) group.

### Lipidomic profiling

Pre-treatment serum samples were collected from 12 CRC patients, comprising 4 patients in the R group and 8 patients in the NR group. Widely targeted lipidomic profiling was performed using an ultra-performance liquid chromatography-tandem mass spectrometry (UPLC-MS/MS) system (UPLC, ExionLC AD, https://sciex.com.cn/; MS, QTRAP^®^ 6500 + System, https://sciex.com.cn/) at Wuhan Metware Biotechnology. Briefly, samples were dried and redissolved. Metabolite quantification was analyzed using a liquid chromatography-electrospray ionization-tandem mass spectrometry (LC-ESI-MS/MS) system. UPLC conditions included a Thermo Accucore^™^ C30 column (2.6 μm, 2.1 mm × 100 mm) with a solvent system of [A: acetonitrile/water (60/40, v/v) (0.1% formic acid, 10 mmol/L ammonium formate)] and [B: acetonitrile/isopropanol (10/90, v/v, 0.1% formic acid, 10 mmol/L ammonium formate)]. The gradient program was as follows: 80:20(v/v) A/B at 0 min, 70:30 (v/v) at 2 min, 40:60 (v/v) at 4 min, 15:85 (v/v) at 9 min, 10:90 (v/v) at 14 min, 5:95 (v/v) at 15.5 and 17.3 min, returning to 80:20 (v/v) at 17.3 min and 20 min. The flow rate was 0.35 ml/min, column temperature was 45 °C, and injection volume was 2 μL. The effluent was connected to an ESI-QTRAP-MS. After quality control of the samples, a total of 1275 lipid metabolites were detected.

### Bioinformatics and data analysis

Statistical analyses and graph generation were performed using R software (version 4.1.1). Variable importance in projection (VIP) scores was calculated *via* orthogonal partial least squares discriminant analysis (OPLS-DA). Differential lipid metabolites between NR and R groups were identified based on the following criteria: |log_2_FC| ≥ 1.5, and *p* < 0.05. Metabolites were annotated using the Kyoto Encyclopedia of Genes and Genomes (KEGG) Compound database and mapped to KEGG Pathway database (http://www.kegg.jp/kegg/pathway.html). Significantly regulated metabolites underwent Metabolite Set Enrichment Analysis (MSEA), with pathway significance determined by hypergeometric test *P*-values.

### Statistical analysis

The associations between BMI and the baseline characteristics of the patients or ORR were analyzed by the χ^2^ test or Fisher’s exact test. PFS and OS were estimated by the Kaplan–Meier method and compared with the log-rank test. A multivariate Cox proportional hazards model was performed to assess the impact of covariates on PFS. The regression model was tested for multicollinearity using variance inflation factor (VIF), A VIF >10 is regarded as indicating serious multicollinearity [20]. All statistical tests were two-sided, with *p* < 0.05 considered statistically significant. Statistical analyses were carried out in Statistical Package for the Social Sciences (SPSS) version 22.0 (IBM, Armonk, NY, USA).

## Results

### Characteristics of CRC patient treated with anti-PD-1 therapy

**Figure 1.**
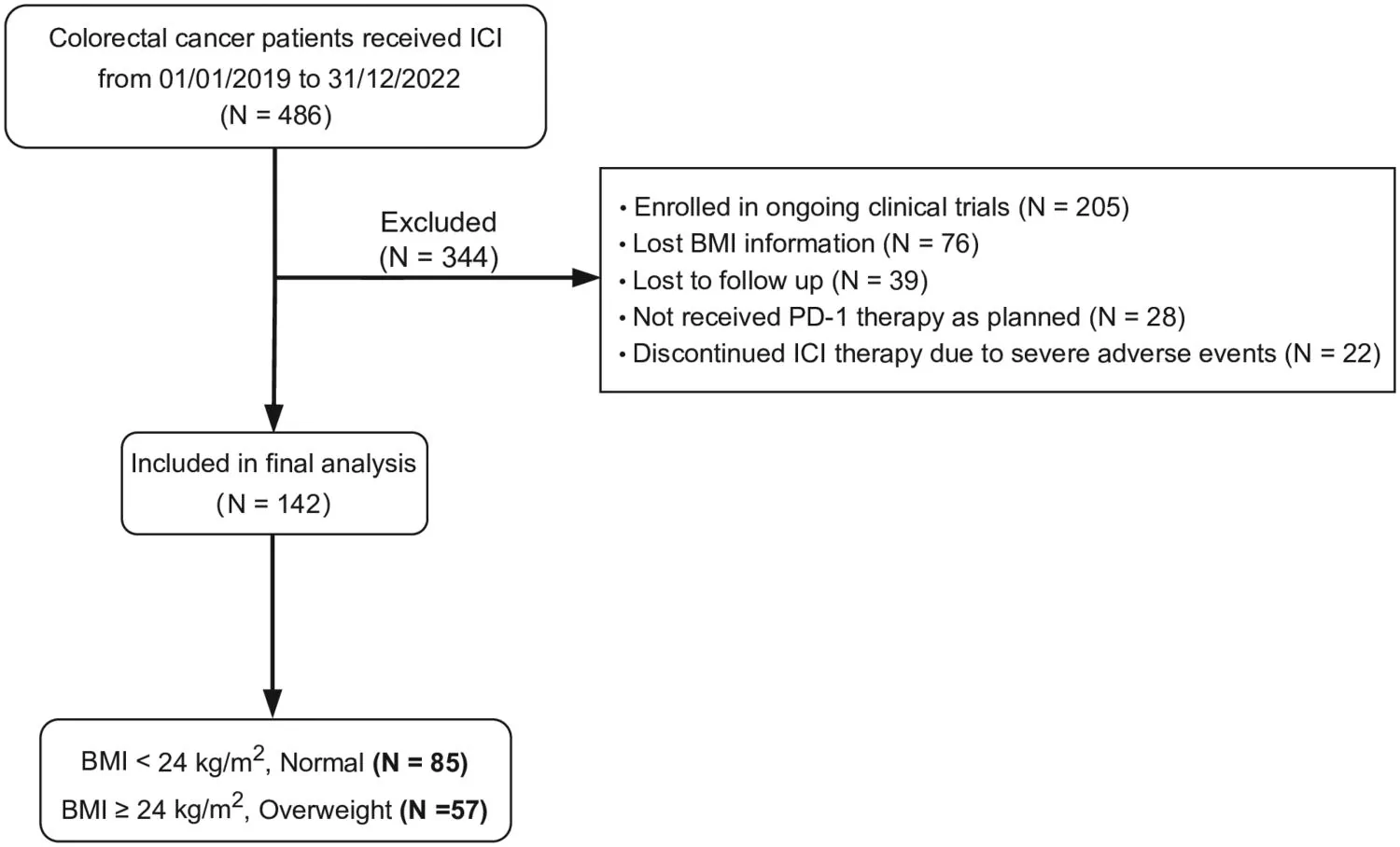
Flowchart illustrating the participant selection process and applied exclusion criteria.

From January 1, 2019, to December 31, 2022, a total of 486 colorectal cancer (CRC) patients treated with ICIs therapy at Sun Yat-sen University Cancer Center (SYSUCC) were retrospectively identified. After applying exclusion criteria, 142 patients with advanced or metastatic CRC who underwent surgical resection were enrolled in the final analysis cohort (Figure 1). All patients completed at least two cycles of anti-PD-1 therapy, with 47 patients (33.1%) achieving a complete response. Based on BMI classification criteria 85 patients (59.9%) were classified as normal weight and 57 patients (40.1%) as overweight. Dyslipidemia, defined by metabolic thresholds predefined in the method, was observed in 70 patients (49.3%). Key baseline characteristics included male sex (67.6%), ECOG performance status of 0 (68.3%), no comorbidities (81.7%), MSI-H status (79.6%), no JAK1/2 mutation (85.7%), no B2M mutant (79.8%), adenocarcinoma histology (88.0%), clinical stage II/III disease (57.7%), no previous treatment (61.3%) and PD-1 monotherapy (46.5%). Tumor location was balanced, with 73 patients (51.4%) presenting with left-sided tumors and 69 patients (48.6%) presenting with right-sided tumors. Comprehensive demographic and clinical data are summarized in Table 1.

**Table 1.** Clinicopathological characteristics of the CRC patients.

		No. (%)
Age		
	< 45	69 (48.6)
	≥ 45	73 (51.4)
Sex		
	Male	96 (67.6)
	Female	46 (32.4)
ECOG		
	0	97 (68.3)
	≥ 1	45 (31.7)
Comorbidity		
	No	116 (81.7)
	Hypertension	19 (13.4)
	Diabetes	4 (2.8)
	Hypothyroidism	2 (1.4)
	Hyperuricemia	1 (0.7)
BMI		
	< 24	85 (59.9)
	≥ 24	57 (40.1)
Dyslipidemia		
	No	72 (50.7)
	Yes	70 (49.3)
Histology		
	Adenocarcinoma	125 (88.0)
	Mucinous	17 (12.0)
Tumor location		
	Left-sided	73 (51.4)
	Right-sided	69 (48.6)
TNM Stage		
	II-III	82 (57.7)
	IV	60 (42.3)
MSI status		
	MSS	29 (20.4)
	MSI-H	113 (79.6)
JAK1/2 mutant^a^		
	No	72 (85.7)
	Yes	12 (14.3)
B2M mutant^a^		
	No	67 (79.8)
	Yes	17 (20.2)
Previous treatment		
	No	87 (61.3)
	Yes	55 (38.7)
Treatment regimen		
	PD-1 Monotherapy	66 (46.5)
	PD-1 + Chemotherapy	65 (45.8)
	PD-1 + CRT	11 (7.7)
Clinical response		
	Non-CR	95 (66.9)
	CR	47 (33.1)

^a^58 patients were not assessed for these mutations due to lack of NGS data.

ECOG: Eastern Cooperative Oncology Group; BMI: Body Mass Index; TNM: tumor-node-metastasis; MSS: Microsatellite Stable; MSI-H: microsatellite instability-high; CRT: Chemoradiotherapy; CR: Complete Response.

Subsequently, we compared the normal weight and overweight groups. The results indicated no statistically significant differences between the overweight and normal weight groups in terms of age, sex, ECOG performance status, comorbidities, histology, tumor location, TNM stage, MSI status, JAK1/2 mutant, B2M mutant, previous treatment, treatment regimen and clinical response (Table 2). Consistent with expectations, the prevalence of dyslipidemia was significantly higher in the overweight group compared with the normal weight group (*p* = 0.001). Furthermore, we performed a subgroup analysis of MSI-H patients. The baseline clinicopathological characteristics of the 113 MSI-H CRC patients are summarized in Table S1. We also performed a subgroup analysis stratified by BMI (Table S2). Compared to the normal weight group, the overweight group had higher proportions of patients with dyslipidemia (*p* = 0.004), complete response (*p* = 0.031) and a lower proportion of patients with stage IV disease (*p* = 0.024).

**Table 2.** Clinicopathological characteristics of the CRC patients stratified by BMI.

	BMI < 24	BMI ≥ 24	*P*
Age			0.13
< 45 years	46 (66.7)	23 (33.3)	
≥ 45 years	39 (53.4)	34 (46.6)	
Sex			0.27
Male	54 (56.2)	42 (43.8)	
Female	31 (67.4)	15 (32.6)	
ECOG			0.28
0	55 (56.7)	42 (43.3)	
≥1	30 (66.7)	15 (33.3)	
Dyslipidemia			***0.001***
No	53 (73.6)	19 (26.4)	
Yes	32 (45.7)	38 (54.3)	
Comorbidity			0.256
No	72 (62.1)	44 (37.9)	
Yes	13 (50.0)	13 (50.0)	
Histology			0.79
Adenocarcinoma	74 (59.2)	51 (40.8)	
Mucinous	11 (64.7)	6 (35.3)	
Tumor location			0.23
Left-sided	40 (54.8)	33 (45.2)	
Right-sided	45 (65.2)	24 (34.8)	
TNM Stage			0.086
II-III	44 (53.7)	38 (46.3)	
IV	41 (68.3)	19 (31.7)	
MSI status			0.67
MSS	16 (55.2)	13 (44.8)	
MSI-H	69 (61.1)	44 (38.9)	
JAK1/2 mutant			0.184
No	42 (58.3)	30 (41.7)	
Yes	10 (83.3)	2 (16.7)	
B2M mutant			0.790
No	41 (61.2)	26 (38.8)	
Yes	11 (64.7)	6 (35.3)	
Previous treatment			
No	52 (59.8)	35 (40.2)	0.978
Yes	33 (60.0)	22 (40.0)	
Treatment regimen			
PD-1 Monotherapy	40 (60.6)	26 (39.4)	0.930
PD-1 + Chemotherapy	39 (60.0)	26 (40.0)	
PD-1 + CRT	6 (54.5)	5 (45.5)	
Clinical response			0.071
Non-CR	62 (65.3)	33 (34.7)	
CR	23 (48.9)	24 (51.1)	

BMI: Body Mass Index; ECOG: Eastern Cooperative Oncology Group; TNM: tumor-node-metastasis; MSS: Microsatellite Stable; MSI-H: microsatellite instability-high; CRT: Chemoradiotherapy; CR: Complete Response. Bold values indicate statistical significance (*p* < 0.05)

### Prognostic impact of BMI and dyslipidemia in anti-PD-1-treated CRC

**Figure 2.**
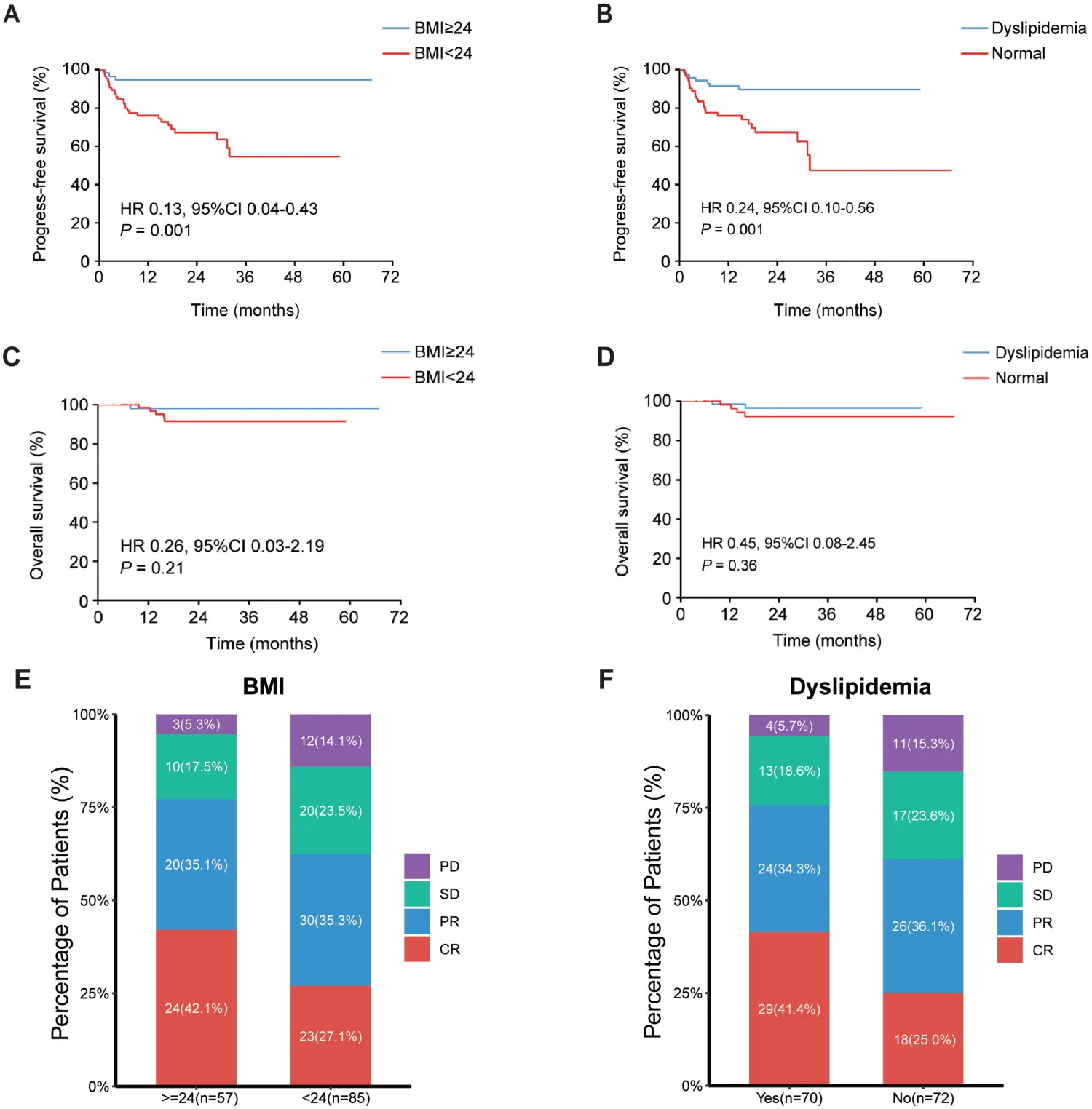
Kaplan-Meier curves depicting progression-free survival in CRC patients. (A, B) Progression-free survival (PFS) stratified by body mass index (BMI) and lipid status. (C, D) Overall survival (OS) stratified by BMI and lipid status. (E, F) Comparison of Best Overall Response (BOR) rates by BMI and dyslipidemia.

First, the prognostic value of BMI in CRC patients received anti-PD-1 therapy was evaluated. PFS was significantly longer in overweight patients (HR 0.13, 95% CI 0.04–0.43, *p* = 0.001) compared with those with normal BMI (Figure 2A). Given the strong correlation between BMI and dyslipidemia reported in multiple studies [21], we further analyzed the prognostic value of lipid abnormalities. Similarly, patients with dyslipidemia exhibited prolonged PFS (HR 0.24, 95% CI 0.10–0.56, *p* = 0.001, Figure 2B). However, no statistically significant differences in overall survival (OS) were observed between groups with different BMI or dyslipidemia status (Figure 2C, D). The median follow-up duration was 23.2 months. Notably, the cohort exhibited a high survival rate, with 95.8% (136/142) of patients censored at the end of the study and only 4.2% (6/142) death events recorded. Patients with high BMI demonstrated a more favorable Best Overall Response (BOR) profile, characterized by higher CR rates (42.1% vs. 27.1%) and lower PD rates (5.3% vs. 14.1%) compared to the normal BMI group (Figure 2E). In the dyslipidemia cohort, we observed superior efficacy outcomes compared to the non-dyslipidemia group (Figure 2F), driven by an increased CR rate (41.4% vs. 25.0%) and a reduced incidence of PD (5.7% vs. 15.3%). In addition, we observed the same findings in the MSI-H subgroup, where patients with higher BMI and dyslipidemia also exhibited longer PFS (HR 0.07, 95% CI 0.01–0.54, *p* = 0.01 and HR 0.1, 95% CI 0.02–0.44, *p* = 0.002, respectively, Figure S1A, B).

Building on our prior analysis of JAK1/2 or B2M mutations in predicting PD-1 outcomes [22], we sought to determine whether tumor mutations could influence the predictive value of BMI and dyslipidemia. A subgroup of 84 patients with available tumor sequencing was analyzed. In this subgroup, among 58 patients (69%) with JAK1/2 and B2M wild-type tumors, Kaplan-Meier analysis showed significant PFS differences based on BMI stratification: overweight patients exhibited longer PFS than normal-weight patients (*p* = 0.008, Figure S2A). In contrast, no significant association between BMI and outcomes was observed in patients with JAK/B2M-mutant tumors (*p* = 0.49, Figure S2B).

### Univariate and multivariate cox regression analysis

Univariate and multivariate Cox regression analyses were performed to reveal significant associations between clinicopathological variables and PFS. In the univariate analysis, high BMI, dyslipidemia, CR, and MSI-H status were significantly associated with prolonged PFS, whereas stage IV and mucinous histology were associated with shorter PFS (Table 3). After adjusting for variables with *p* < 0.1 in the multivariate analysis, high BMI remained a strong independent prognostic factor for PFS (HR 0.2, 95% CI 0.058–0.69, *p* = 0.011). Additionally, TNM stage (*p* = 0.006), MSI status (*p* = 0.013), histology (*p* = 0.026), and dyslipidemia (*p* = 0.039) were also identified as independent predictors of PFS (Table 4). The Schoenfeld residuals tests demonstrated that the proportional hazards assumption was met for all covariates in the multivariate model (Figure S3A). Additionally, there was no evidence of multicollinearity, as VIF ranged from 1.09 to 1.48 (Figure S3B).

**Table 3.** Univariate analysis of factors associated with progression-free survival.

Variables	Progression free survival	
	HR (95%CI)	*P*
Sex		
Female	reference	
Male	0.78 (0.38–1.64)	0.52
Age		
< 45 years	reference	
≥ 45 years	0.97 (0.48–1.98)	0.94
ECOG		
0	reference	
≥1	1.76 (0.86–3.60)	0.12
BMI		
< 24	reference	
≥ 24	0.13 (0.04–0.43)	***0.001***
Dyslipidemia		
No	reference	
Yes	0.24 (0.10–0.56)	***0.001***
Tumor location		
Left-sided	reference	
Right-sided	1.03 (0.51–2.07)	0.95
TNM Stage		
II-III	reference	
IV	8.28 (3.18–21.58)	***0.000***
Histology		
Adenocarcinoma	reference	
Mucinous	5.30 (2.54–11.09)	***0.000***
Clinical response		
Non-CR	reference	
CR	0.024 (0.001–0.51)	***0.017***
MSI status		
MSS	reference	
MSI-H	0.29 (0.14–0.61)	***0.001***

BMI: Body Mass Index; ECOG: Eastern Cooperative Oncology Group; TNM: tumor-node-metastasis; CR: Complete Response; MSS: Microsatellite Stable; MSI-H: microsatellite instability-high. Bold values indicate statistical significance (*p* < 0.05)

**Table 4.** Multivariate cox regression analysis of factors associated with progression-free survival.

Variable	HR (95% CI)	*P*
BMI (≥ 24 vs. < 24)	0.20 (0.058–0.69)	***0.011***
TNM Stage (IV vs. II-III)	4.15 (1.50–11.46)	***0.006***
MSI status (MSI-H vs. MSS)	0.37 (0.17–0.81)	***0.013***
Histology (Mucinous vs. Adenocarcinoma)	2.44 (1.11–5.33)	***0.026***
Dyslipidemia (Yes vs. No)	0.40 (0.16–0.96)	***0.039***

Schoenfeld residuals suggested that the Cox models met proportional hazards assumptions.

BMI: Body Mass Index; TNM: tumor-node-metastasis; MSI-H: microsatellite instability-high; MSS: Microsatellite Stable. Bold values indicate statistical significance (*p* < 0.05)

### Association between BMI and irAEs in CRC patients treated with ICIs

In our cohort, 57 (40.1%) patients developed any-grade immune-related adverse events (irAEs). The incidence of irAEs was comparable between the high BMI group (46.6%) and the normal weight group (53.4%). No statistically significant association was found between high BMI and the occurrence of irAEs (*p* = 0.696) (Table 5). Furthermore, we characterized organ-specific toxicities related to irAEs based on clinical records (Table S3). The most frequently observed toxicities were rash and increased aminotransferase. Owing to the low overall incidence of grade 3–4 events, the statistical power for analyzing high-grade toxicities was limited, therefore, these events were only summarized descriptively and were not included in further correlative analyses.

**Table 5.** The association between the incidence of any-grade immune-related adverse events andz body mass index categories.

	BMI < 24	BMI ≥ 24	*P*
IrAE status			0.696
No	52 (66.7)	33 (33.3)	
Yes	33 (53.4)	24 (46.6)	
IrAE level			0.346
Grade1-2	24 (56.2)	20 (43.8)	
Grade3-4	9 (67.4)	4 (32.6)	

irAE: immune-related adverse event.

### Lipid metabolites influence CRC patients’ response to anti-PD-1 therapy

**Figure 3.**
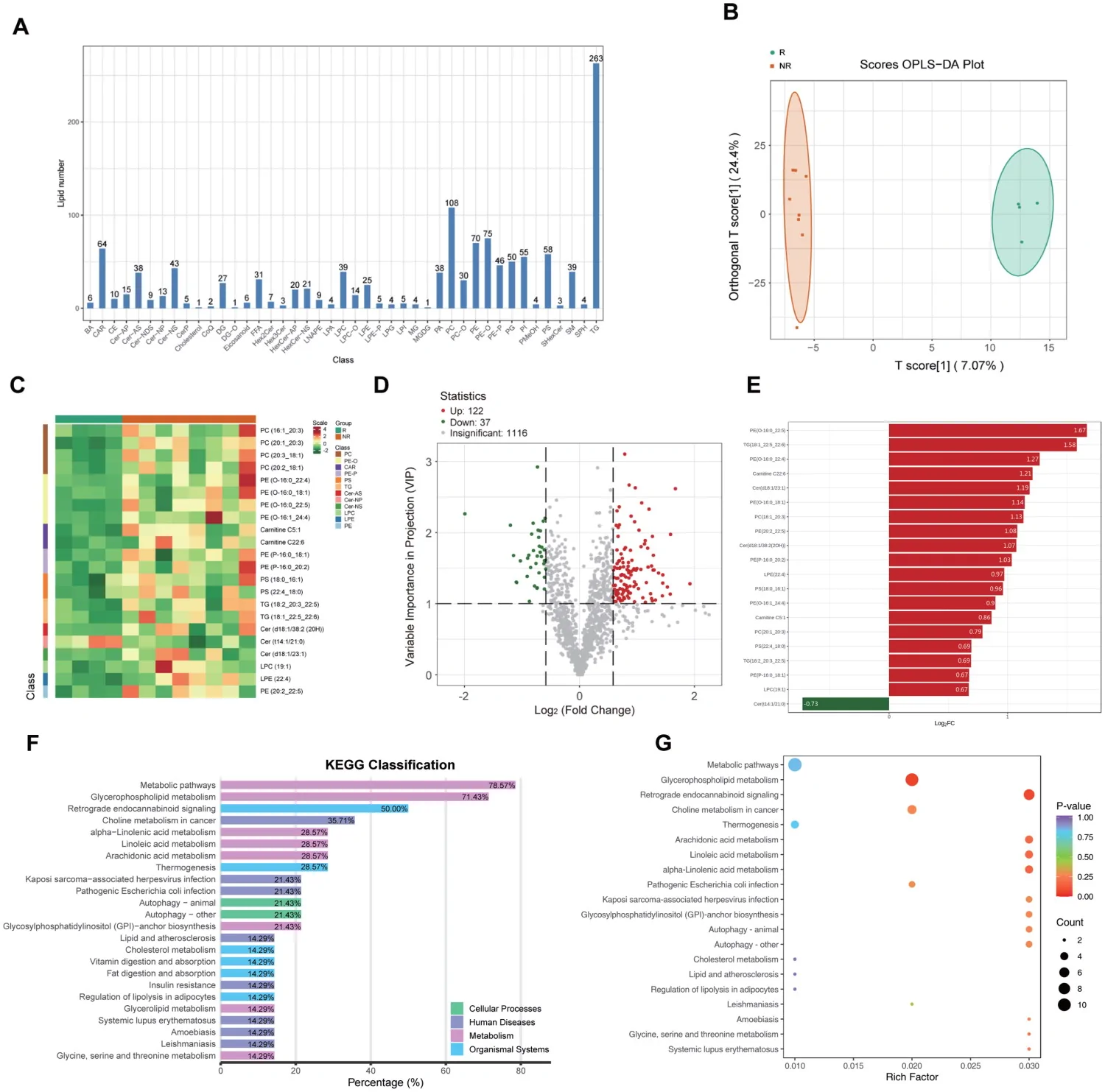
Baseline serum lipidomic profiling reveals distinct metabolic signatures associated with tumor recurrence. (A) Bar chart displaying the total number of lipid species identified across different lipid classes, with Triglycerides (TG) and Phosphatidylcholines (PC) being the most abundant. (B) Distinct lipidomic profiles based on clinical outcome. OPLS-DA score plot showing a clear separation between patients with Recurrence (R, *n* = 4) and those with Non-Recurrence (NR, *n* = 8) group. The distinct clustering indicates that systemic lipid metabolism is altered between R and NR group. (C) Heatmap of differential lipids. Hierarchical clustering heatmap visualizing the relative abundance of the top differentially expressed lipids. Rows represent individual lipid species and columns represent samples. (D) Identification of key biomarkers (NR VS R). Volcano plot identifying 22 significantly altered lipids (|log_2_FC| ≥ 1.5 and *p* < 0.05). Red and green dots indicate upregulated (*n* = 21) and downregulated (*n* = 1) lipids in the NR group, respectively. (E) Top 20 differential metabolites (NR VS R). LDA effect size analysis of the top 20 differential metabolites between the NR and R groups. (F) KEGG classification bar chart categorizing the differentially expressed lipids into biological processes. The majority of altered lipids are mapped to metabolic pathways and glycerophospholipid metabolism. (G) KEGG pathway enrichment analysis (bubble plot). The x-axis represents the Rich Factor (ratio of differential lipids to total lipids in the pathway), and the y-axis represents the pathway name. Dot size indicates the count of differential metabolites, and color indicates statistical significance (p-value).

To further investigate the metabolic underpinnings of this association, we performed lipidomic profiling of serum samples to identify key lipid metabolites involved in CRC progression following anti-PD-1 therapy. Pre-treatment serum samples from 12 CRC patients (4 patients in the R group and 8 patients in the NR group) were subjected to lipidomic analysis by UPLC-MS/MS. Quantitative analysis of lipids identified 1,275 lipid metabolites and 43 lipid subclasses. Triglycerides (TG) constituted the most abundant subclass (20.63%, 263 molecules), followed by phosphatidylcholine (PC, 8.47%, 108 molecules) and phosphatidylethanolamine ethers (PE-O, 5.88%, 75 molecules, Figure 3A). The OPLS-DA score plot was utilized to visualize the overall lipidomic distributions. As illustrated in Figure 3B, a clustering trend was observed between the R and NR groups, suggesting distinct lipid metabolic phenotypes.

Differential lipid heatmap analysis showed marked downregulation of PC, PE, and carnitine in the R group compared with the NR group (Figure 3C). These molecules are critical for cellular activity [10]. After applying thresholds (|log_2_FC| ≥ 1.5, *p* < 0.05), 23 differentially abundant lipid metabolites were identified: 22 upregulated and 1 downregulated in the NR group compared to the R group (Figure 3D). The top 20 differential metabolites (Figure 3E) were predominantly enriched in the NR group and many of them were associated with immune reprogramming and signaling pathways.

Subsequently, KEGG pathway analysis highlighted significant enrichment of lipid metabolic pathways in the NR group, particularly those involving glycerophospholipid metabolism, alpha-linolenic acid metabolism, linoleic acid metabolism, and arachidonic acid metabolism (Figure 3F, G). Full statistical details, including ratios and adjusted p-values for all enriched pathways, are provided in Table S4. Collectively, these metabolic pathways likely play a critical role in anti-PD-1 therapy response in CRC patients, a finding that warrants further investigation.

## Discussion

Our study provides the first demonstration that elevated pre-treatment BMI (≥ 24 kg/m^2^) predicts improved PFS in CRC patients treated with anti-PD-1 therapy, offering robust clinical evidence supporting the debated ‘obesity paradox’. Furthermore, dyslipidemia was also identified as an independent prognostic factor, implying that systemic lipid homeostasis may modulate ICIs efficacy. Mechanistically, this association with enhanced antitumor immunity appears to be mediated by obesity-related lipid metabolic reprogramming, as supported by our lipidomic analysis, which revealed distinct glycerophospholipid metabolism profiles between R and NR groups.

Accumulating evidence consistently links elevated BMI to improved immunotherapeutic outcomes across various malignancies, including endometrial cancer, NSCLC and melanoma [23–25]. A large pan-cancer cohort further confirmed that obese patients (BMI ≥ 30) exhibit superior response rates and survival [26]. Consistent with these findings, our study observed a similar trend. However, the absence of significant OS difference in our cohort may be due to the low number of death events, which limited the statistical power. Notably, our study adopted the WHO-recommended BMI classification for Chinese populations. This methodological distinction underscores the potential of standardized BMI criteria to improve the generalizability of findings across diverse ethnic groups.

Current literature regarding the impact of obesity on tumor immunity is conflicting. Substantial evidence suggests that obesity fosters an immunosuppressive microenvironment that facilitates tumor progression. For instance, obesity has been shown to drive the accumulation of myeloid-derived suppressor cells (MDSCs) [27] and induce T-cell dysfunction *via* adipocyte-derived extracellular vesicles [28], seemingly contradicting a survival benefit. However, we hypothesize that this specific immunosuppressive landscape paradoxically primes the tumor for improved responsiveness to PD-1 blockade. While obesity-induced metabolic stress impairs T-cell function, recent mechanistic studies reveal that this manifests as reversible functional inhibition (characterized by high PD-1 expression) rather than terminal exhaustion [29]. Consequently, these ‘paused’ T cells remain amenable to reactivation [11]. This is particularly relevant in the context of obesity, which induces PD-1 upregulation and may render tumors highly dependent on this inhibitory axis. The impact of this mechanism is likely amplified in our MSI-H predominant cohort. Given that MSI-H tumors are inherently characterized by a hyperactive immune microenvironment with abundant T-cell infiltration, the removal of the obesity-mediated PD-1 brake may thus effectively unleash pre-existing anti-tumor response.

Given the association between BMI and lipid metabolism, dyslipidemia may partially mediate the impact of obesity on the tumor microenvironment. Mechanistically, elevated lipids can drive T-cell exhaustion and directly upregulate PD-1 expression *via* cholesterol-induced ER stress [30–32]. However, our multivariate analysis (VIF < 2) indicates that BMI retains prognostic value independent of lipid levels. This independence is exemplified in patients with ‘metabolically healthy obesity’ (high BMI with normal lipids) [33], where expanded adipose tissue exerts immunomodulatory effects *via* leptin. Leptin promotes an inflammatory phenotype by suppressing Tregs and enhancing IFN-γ-mediated PD-L1 upregulation [34,35]. Collectively, whether through lipid-driven metabolic exhaustion (leading to high PD-1) or leptin-driven inflammatory activation (leading to high PD-L1), these concurrent mechanisms synergistically create an immune microenvironment highly sensitive to PD-1 blockade.

Metabolic reprogramming of fatty acids (FAs) is pivotal for immune regulation. FA synthesis drives T-cell proliferation, while fatty acid oxidation (FAO) fuels memory T-cell survival [36–39]. In our study, the NR group exhibited significant upregulation of 21 lipid metabolites, predominantly glycerophospholipids. Biologically, this enrichment likely boosts anti-tumor immunity through two key mechanisms. First, glycerophospholipids optimize membrane dynamics and facilitate Phospholipase C gamma1 (PLCγ1) recruitment, thereby enhancing T-cell receptor signaling and effector function—a phenotype previously linked to ICIs response [40]. Second, in the glucose-deprived tumor microenvironment, abundant lipids serve as critical fuel for FAO-driven T-cell persistence [41]. Collectively, this lipid-rich profile reflects a metabolic adaptation that sustains T-cell activation, synergizing with PD-1 blockade to improve clinical outcomes.

Certain limitations in our study warrant acknowledgment. First, the BMI cutoff (≥ 24 kg/m^2^) adopted in this study is specific to the Chinese population, necessitating validation in diverse ethnic groups. Second, regarding the subgroup analyses stratified by JAK1/2 and B2M status, due to the limited sample sizes of the mutant subgroups, these analyses may lack sufficient statistical power. Third, the sample size for lipidomic profiling (*n* = 12) was relatively small. To avoid missing potential signals in this pilot phase, our lipidomic analysis prioritized nominal p-values over stringent FDR correction. While this strategy successfully identified biologically relevant pathways consistent with clinical outcomes, the resulting OPLS-DA models and pathway enrichments should be considered exploratory. Finally, the underlying biological mechanisms remain to be fully characterized. Future multicenter cohorts are required to validate these preliminary signals and enhance the external validity of our conclusions.

In conclusion, our study positions BMI as a valuable prognostic biomarker for CRC patients undergoing anti-PD-1 immunotherapy. The observed metabolic alterations associated with obesity appear to functionally enhance immunotherapy efficacy, potentially mediated through obesity-modulated immune-metabolic interactions. Future research should focus on integrating clinical observations with mechanistic investigations to develop precision-based, metabolism-informed strategies for optimizing immunotherapy outcomes in CRC management.

## Supplementary Material

Supplemental Material

Supplemental Material

Supplemental Material

## Data Availability

The datasets used and/or were analyzed during the current study are available from the corresponding author on reasonable request.
