# Supplementary material for: Body mass index and dyslipidemia influence responses to anti-PD-1 immunotherapy in colorectal cancer
Source: Ann Med. 2026 Aug 24;58(1):2624219. doi: 10.1080/07853890.2026.2624219 (PMC13520866; doi:10.1080/07853890.2026.2624219)
Supplement: Supplementary file 1 [file IANN_A_2624219_SM9568.pdf]

Figure1

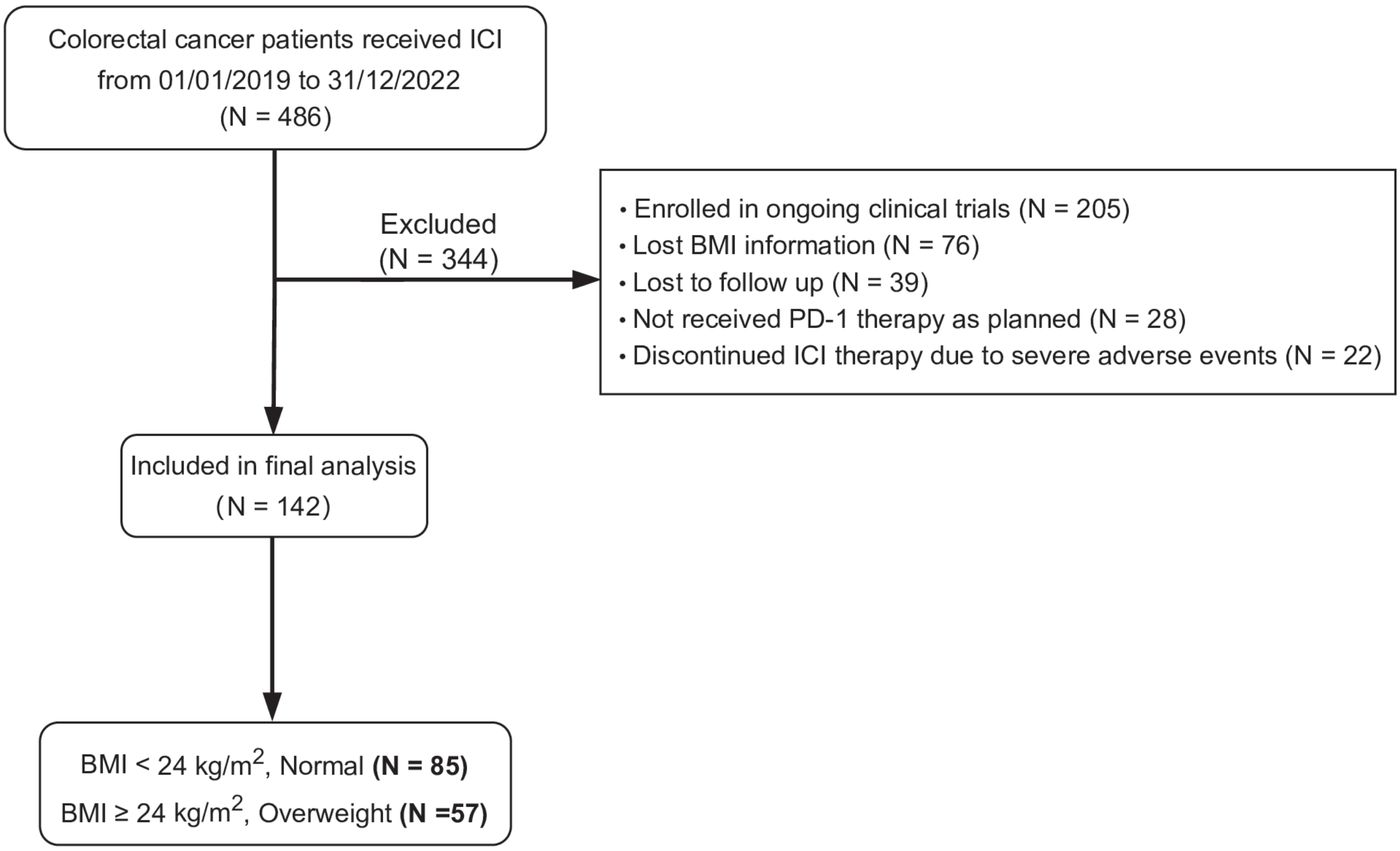

Colorectal cancer patients received ICI
from 01/01/2019 to 31/12/2022
(N = 486)
Excluded
(N = 344)
• Enrolled in ongoing clinical trials (N = 205)
• Lost BMI information (N = 76)
• Lost to follow up (N = 39)
• Not received PD-1 therapy as planned (N = 28)
• Discontinued ICI therapy due to severe adverse events (N = 22)
Included in final analysis
(N = 142)
BMI < 24 kg/m2, Normal (N = 85)
BMI ≥ 24 kg/m2, Overweight (N =57)

Figure 2

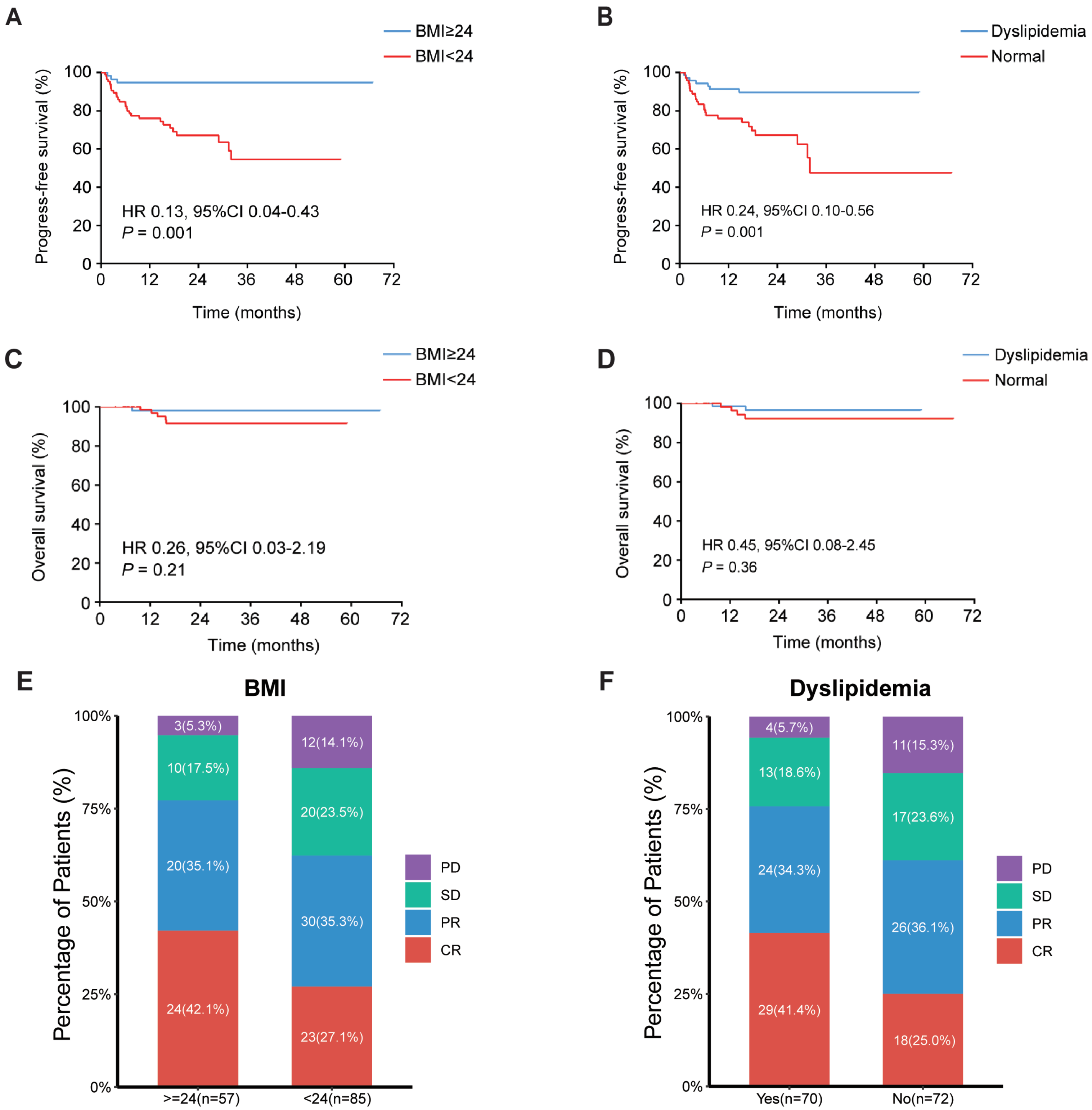
A
BMI≥24
BMI<24
Progress-free survival (%)
HR 0.13, 95%CI 0.04-0.43
P = 0.001
Time (months)
B
Dyslipidemia
Normal
Progress-free survival (%)
HR 0.24, 95%CI 0.10-0.56
P = 0.001
Time (months)
C
BMI≥24
BMI<24
Overall survival (%)
HR 0.26, 95%CI 0.03-2.19
P = 0.21
Time (months)
D
Dyslipidemia
Normal
Overall survival (%)
HR 0.45, 95%CI 0.08-2.45
P = 0.36
Time (months)
E
BMI
Percentage of Patients (%)
3(5.3%)
10(17.5%)
20(35.1%)
24(42.1%)
12(14.1%)
20(23.5%)
30(35.3%)
23(27.1%)
>=24(n=57)
<24(n=85)
PD
SD
PR
CR
F
Dyslipidemia
Percentage of Patients (%)
4(5.7%)
13(18.6%)
24(34.3%)
29(41.4%)
11(15.3%)
17(23.6%)
26(36.1%)
18(25.0%)
Yes(n=70)
No(n=72)
PD
SD
PR
CR

Figure 3

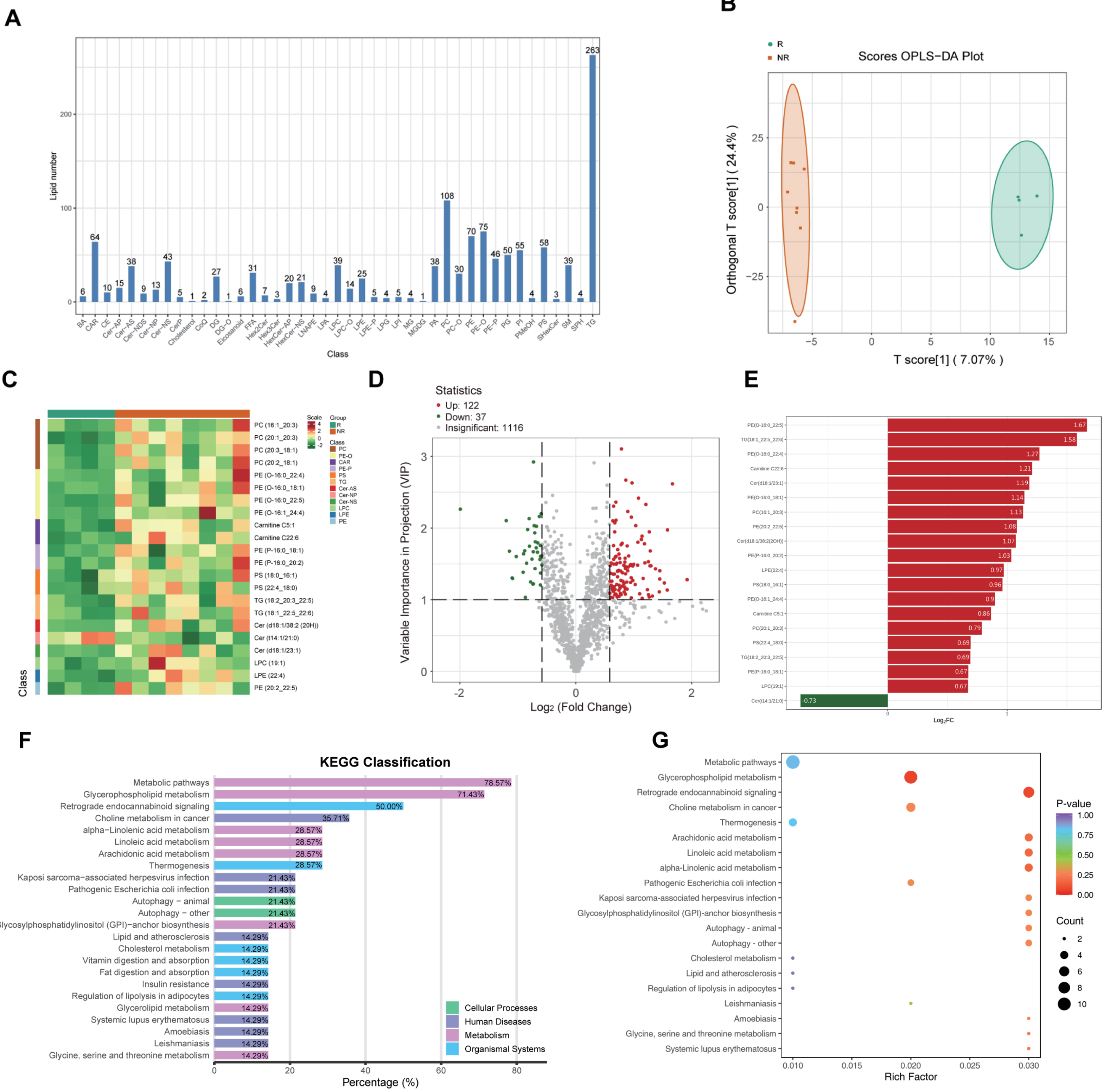
A
Lipid number
Class
BA
CAR
CE
Cer-AP
Cer-AS
Cer-NDS
Cer-NP
Cer-NS
CerP
Cholesterol
CoQ
DG
DG-O
Eicosanoid
FFA
Hex2Cer
Hex3Cer
HexCer-AP
HexCer-NS
LNAPE
LPA
LPC
LPC-O
LPE
LPE-P
LPG
LPI
MG
MGDG
PA
PC
PC-O
PE
PE-O
PE-P
PG
PI
PMeOH
PS
SHexCer
SM
SPH
TG
B
R
NR
Scores OPLS−DA Plot
Orthogonal T score[1] (24.4%)
T score[1] (7.07%)
C
Class
Scale
Group
PC (16:1_20:3)
PC (20:1_20:3)
PC (20:3_18:1)
PC (20:2_18:1)
PE (O-16:0_22:4)
PE (O-16:0_18:1)
PE (O-16:0_22:5)
PE (O-16:1_24:4)
Carnitine C5:1
Carnitine C22:6
PE (P-16:0_18:1)
PE (P-16:0_20:2)
PS (18:0_16:1)
PS (22:4_18:0)
TG (18:2_20:3_22:5)
TG (18:1_22:5_22:6)
Cer (d18:1/38:2 (20H))
Cer (t14:1/21:0)
Cer (d18:1/23:1)
LPC (19:1)
LPE (22:4)
PE (20:2_22:5)
D
Statistics
Up: 122
Down: 37
Insignificant: 1116
Variable Importance in Projection (VIP)
Log2 (Fold Change)
E
Log2FC
F
KEGG Classification
Metabolic pathways
Glycerophospholipid metabolism
Retrograde endocannabinoid signaling
Choline metabolism in cancer
alpha−Linolenic acid metabolism
Linoleic acid metabolism
Arachidonic acid metabolism
Thermogenesis
Kaposi sarcoma−associated herpesvirus infection
Pathogenic Escherichia coli infection
Autophagy − animal
Autophagy − other
Glycosylphosphatidylinositol (GPI)−anchor biosynthesis
Lipid and atherosclerosis
Cholesterol metabolism
Vitamin digestion and absorption
Fat digestion and absorption
Insulin resistance
Regulation of lipolysis in adipocytes
Glycerolipid metabolism
Systemic lupus erythematosus
Amoebiasis
Leishmaniasis
Glycine, serine and threonine metabolism
Cellular Processes
Human Diseases
Metabolism
Organismal Systems
Percentage (%)
G
P-value
Count
Rich Factor

Figure S1

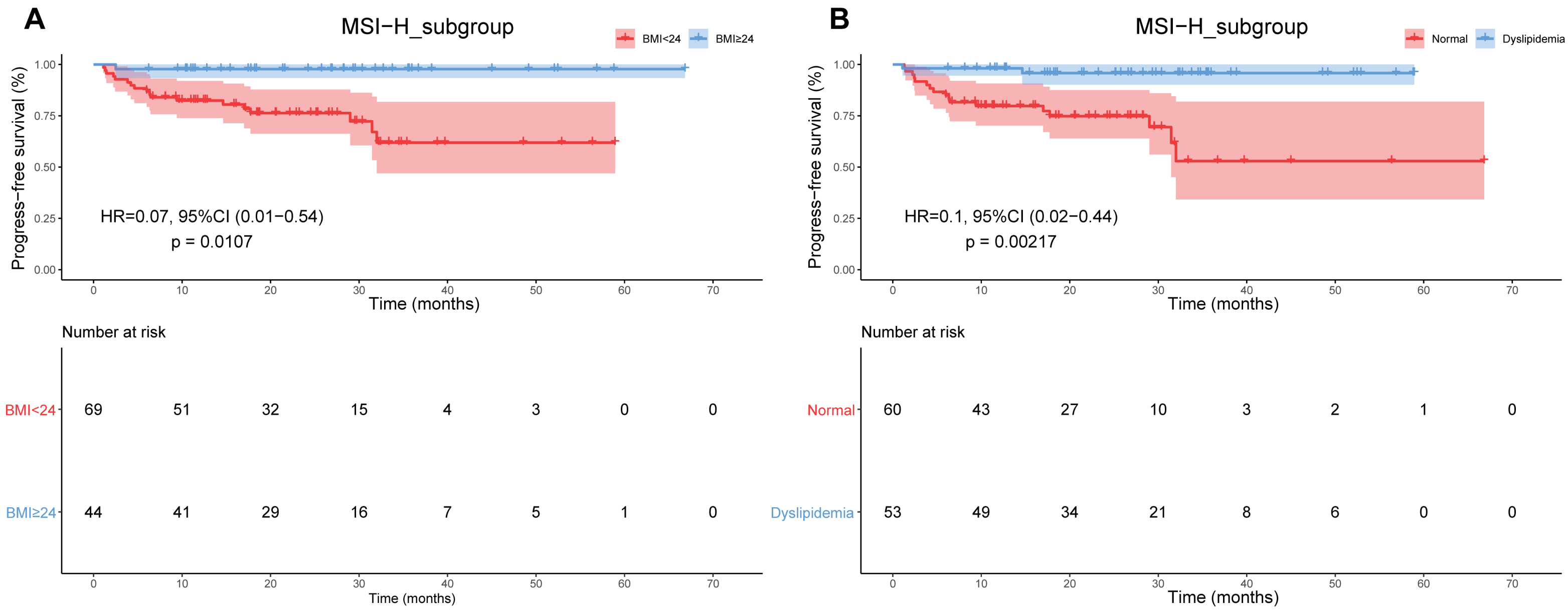
A
MSI−H_subgroup
BMI<24
BMI≥24
Progress−free survival (%)
1.00
0.75
0.50
0.25
0.00
HR=0.07, 95%CI (0.01−0.54)
p = 0.0107
0
10
20
30
40
50
60
70
Time (months)
Number at risk
BMI<24 69 51 32 15 4 3 0 0
BMI≥24 44 41 29 16 7 5 1 0
Time (months)
B
MSI−H_subgroup
Normal
Dyslipidemia
Progress−free survival (%)
HR=0.1, 95%CI (0.02−0.44)
p = 0.00217
Time (months)
Number at risk
Normal 60 43 27 10 3 2 1 0
Dyslipidemia 53 49 34 21 8 6 0 0
Time (months)

Figure S2

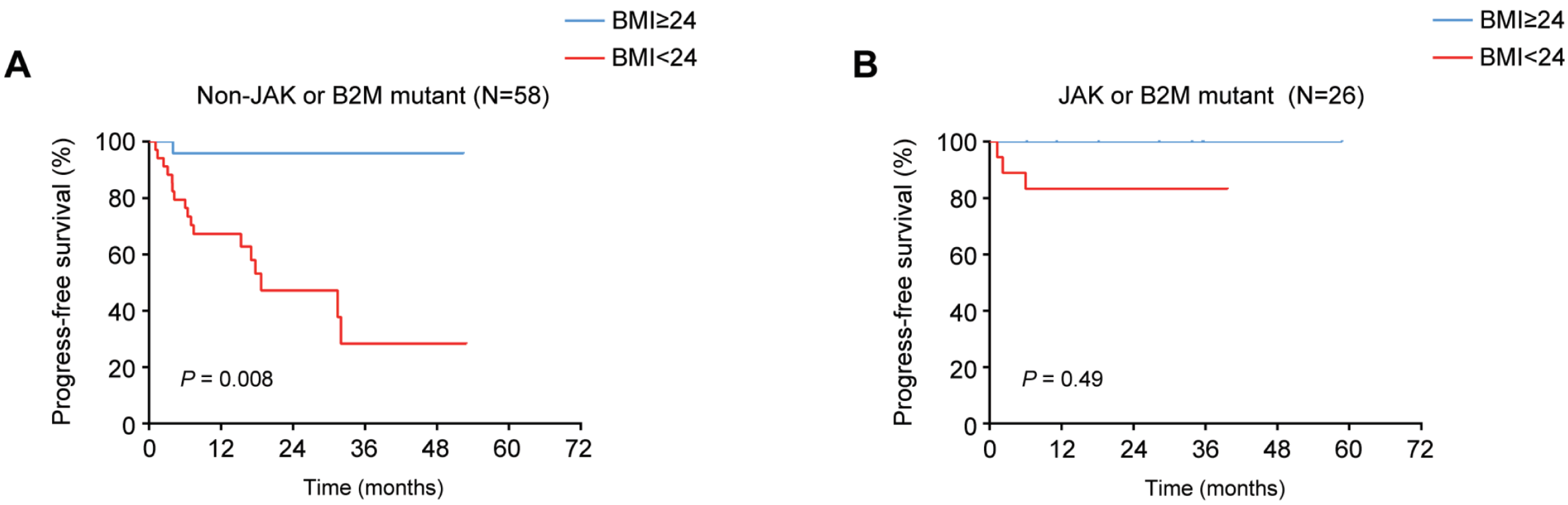
A
BMI≥24
BMI<24
Non-JAK or B2M mutant (N=58)
Progress-free survival (%)
100
80
60
40
20
0
P = 0.008
0
12
24
36
48
60
72
Time (months)
B
BMI≥24
BMI<24
JAK or B2M mutant (N=26)
Progress-free survival (%)
100
80
60
40
20
0
P = 0.49
0
12
24
36
48
60
72
Time (months)

Figure S3

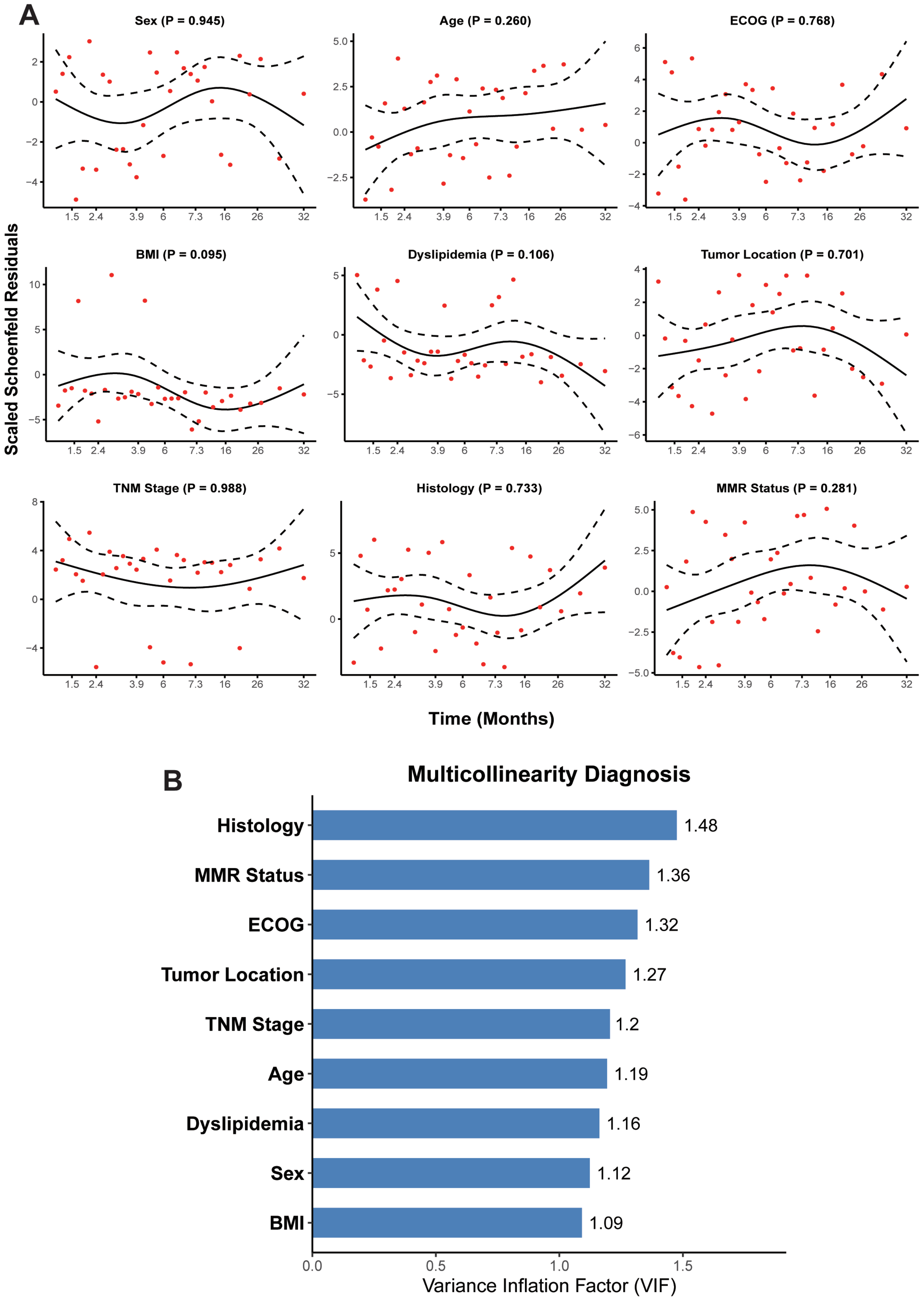

A
Sex (P = 0.945)
Age (P = 0.260)
ECOG (P = 0.768)
BMI (P = 0.095)
Dyslipidemia (P = 0.106)
Tumor Location (P = 0.701)
TNM Stage (P = 0.988)
Histology (P = 0.733)
MMR Status (P = 0.281)
Scaled Schoenfeld Residuals
1.5 2.4 3.9 6 7.3 16 26 32
Time (Months)
B
Multicollinearity Diagnosis
Histology 1.48
MMR Status 1.36
ECOG 1.32
Tumor Location 1.27
TNM Stage 1.2
Age 1.19
Dyslipidemia 1.16
Sex 1.12
BMI 1.09
0.0 0.5 1.0 1.5
Variance Inflation Factor (VIF)
