# Supplementary material for: Body mass index and dyslipidemia influence responses to anti-PD-1 immunotherapy in colorectal cancer
Source: Ann Med. 2026 Aug 24;58(1):2624219. doi: 10.1080/07853890.2026.2624219 (PMC13520866; doi:10.1080/07853890.2026.2624219)
Supplement: Supplementary file 2 [file IANN_A_2624219_SM9565.docx]

**Figure S1.** Progression-free survival (PFS) analysis in the MSI-H subgroup. **(A)** Kaplan-Meier curves of PFS stratified by BMI levels (< 24 vs. ≥ 24) in the MSI-H subgroup. **(B)** Kaplan-Meier curves of PFS comparing patients with normal lipid levels and dyslipidemia in the MSI-H subgroup.

**Figure S2.** Prognostic value of BMI stratified by JAK1/2 or B2M mutation status in CRC patients receiving anti-PD-1 therapy. Kaplan-Meier curves compare progression-free survival by high vs.low BMI in **(A)** patientswithout JAK1/2 and B2M mutations (n=58) and **(B)** patients with JAK1/2 or B2M mutations (n=26).

**Figure S3.** Diagnostic plots for the multivariate Cox proportional hazards regression model. **(A)** Scaled Schoenfeld residual plots confirming that the proportional hazards assumption was met for all covariates (p > 0.05). **(B)** Variance inflation factors (VIF) analysis showing all values were below 2, indicating no significant multicollinearity.
