# Supplementary material for: Body mass index and dyslipidemia influence responses to anti-PD-1 immunotherapy in colorectal cancer
Source: Ann Med. 2026 Aug 24;58(1):2624219. doi: 10.1080/07853890.2026.2624219 (PMC13520866; doi:10.1080/07853890.2026.2624219)
Supplement: Supplementary file 3 [file IANN_A_2624219_SM9634.docx]

**Supplementary Table 1. Clinicopathological characteristics of the MSI-H CRC patients.**

|  | | **No. (%)** |
| --- | --- | --- |
| **Age** | |  |
|  | < 45 | 61 (54.0) |
|  | ≥ 45 | 52 (46.0) |
| **Sex** |  |  |
|  | Male | 78 (69.0) |
|  | Female | 35 (31.0) |
| **ECOG** |  |  |
|  | 0 | 79 (69.9) |
|  | ≥ 1 | 34 (30.1) |
| **BMI** |  |  |
|  | < 24 | 65 (57.5) |
|  | ≥ 24 | 48 (42.5) |
| **Dyslipidemia** | |  |
|  | No | 60 (53.1) |
|  | Yes | 53 (46.9) |
| **Comorbidity**^a^ | |  |
|  | No | 98 (86.7) |
|  | Hypertension | 11 (9.7) |
|  | Diabetes | 2 (1.8) |
|  | Hypothyroidism | 1 (0.9) |
|  | Hyperuricemia | 1 (0.9) |
| **Histology** | |  |
|  | Adenocarcinoma | 100 (88.5) |
|  | Mucinous | 13 (11.5) |
| **Tumor location** | |  |
|  | Left-sided | 56 (49.6) |
|  | Right-Sided | 57 (50.4) |
| **TNM Stage** |  |  |
|  | II-III | 66 (58.4) |
|  | IV | 47 (41.6) |
| **JAK1/2 mutant**^a^ | |  |
|  | No | 53 (82.8) |
|  | Yes | 11 (17.2) |
| **B2M mutant**^a^ | |  |
|  | No | 48 (75.0) |
|  | Yes | 16 (25.0) |
| **Previous treatment** | |  |
|  | No | 79 (69.9) |
|  | Yes | 34 (30.1) |
| **Treatment regimen** |  |  |
|  | PD-1 Monotherapy | 63 (55.8) |
|  | PD-1 + Chemotherapy | 44 (38.9) |
|  | PD-1 + CRT | 6 (5.3) |
|  |  |  |
| **Clinical response** | |  |
|  | Non-CR | 76 (67.3) |
|  | CR | 37 (32.7) |

^a^49 patients were not assessed for these mutations due to lack of NGS data. Abbreviations: ECG, Eastern Cooperative Oncology Group; BMI, Body Mass Index; TNM, tumor-node-metastasis; MSS, Microsatellite Stable; MSI-H, microsatellite instability-high; CRT, Chemoradiotherapy; CR, Complete Response.

**Supplementary Table 2. Clinicopathological characteristics of the MSI-H CRC patients stratified by BMI.**

|  | **BMI <** **24 (N=69)** | **BMI ≥** **24 (N=44)** | ***P*** |
| --- | --- | --- | --- |
| **Age** |  |  | 0.146 |
| < 45 years | 41 (67.2) | 20 (32.8) |  |
| ≥ 45 years | 28 (53.9) | 24 (46.1) |  |
| **Sex** |  |  | 0.673 |
| Male | 23 (63.9) | 13 (36.1) |  |
| Female | 46 (59.7) | 31 (40.3) |  |
| **ECOG** |  |  | 0.643 |
| 0 | 49 (59.8) | 33 (40.2) |  |
| ≥1 | 20 (64.5) | 11 (35.5) |  |
| **Dyslipidemia** | |  | ***0.004*** |
| No | 44 (73.3) | 16 (26.7) |  |
| Yes | 25 (47.2) | 28 (52.8) |  |
| **Comorbidity** | |  | 0.220 |
| No | 62 (63.3) | 36 (36.7) |  |
| Yes | 7 (46.7) | 8 (53.3) |  |
| **Histology** |  |  | 0.463 |
| Adenocarcinoma | 60 (59.4) | 41 (40.6) |  |
| Mucinous | 9 (75.0) | 3 (25.0) |  |
| **Tumor location** |  |  | 0.194 |
| Left-sided | 29 (54.7) | 24 (45.3) |  |
| Right-sided | 40 (66.7) | 20 (33.3) |  |
| **TNM Stage** |  |  | ***0.024*** |
| II-III | 39 (53.4) | 34 (46.6) |  |
| IV | 30 (75.0) | 10 (25.0) |  |
| **JAK1/2 mutant** | |  | 0.316 |
| No | 32 (60.4) | 21 (39.6) |  |
| Yes | 9 (81.8) | 2 (18.2) |  |
| **B2M mutant** | |  | 0.880 |
| No | 31 (64.6) | 17 (35.4) |  |
| Yes | 10 (62.5) | 6 (37.5) |  |
| **Previous treatment** | |  | 0.173 |
| No | 45 (57.0) | 34 (43.0) |  |
| Yes | 24 (70.6) | 10 (29.4) |  |
| **Treatment regimen** |  |  | 0.899 |
| PD-1 Monotherapy | 37 (58.7) | 26 (41.3) |  |
| PD-1 + Chemotherapy | 28 (63.6) | 16 (36.4) |  |
| PD-1 + CRT | 4 (66.7) | 2 (33.3) |  |
| **Clinical response** |  |  | ***0.031*** |
| Non-CR | 47 (69.1) | 21 (30.9) |  |
| CR | 22 (48.9) | 23 (51.1) |  |

Abbreviations: ECOG, Eastern Cooperative Oncology Group; BMI, Body Mass Index; TNM, tumor-node-metastasis; CRT, Chemoradiotherapy; CR, Complete Response.

**Supplementary Table 3. Distribution of organ-specific irAEs stratified by BMI status.**

|  | **BMI <** **24 (N=33)** | **BMI ≥** **24 (N=24)** | ***P*** |
| --- | --- | --- | --- |
| **Constitutional** |  |  |  |
| Fatigue |  |  | - |
| Grade 1-2 | 7 | 2 |  |
| Grade 3-4 | 0 | 0 |  |
| Pyrexia |  |  | - |
| Grade 1-2 | 2 | 5 |  |
| Grade 3-4 | 0 | 0 |  |
| **Skin and mucosa** |  |  |  |
| Rash |  |  | 1.000 |
| Grade 1-2 | 17 | 10 |  |
| Grade 3-4 | 3 | 1 |  |
| Haemangiom |  |  | - |
| Grade 1-2 | 4 | 3 |  |
| Grade 3-4 | 0 | 0 |  |
| Hand and foot syndrome |  |  | - |
| Grade 1-2 | 6 | 3 |  |
| Grade 3-4 | 2 | 0 |  |
| Stomatitis |  |  | - |
| Grade 1-2 | 5 | 2 |  |
| Grade 3-4 | 0 | 0 |  |
| **Gastrointestinal** |  |  |  |
| Abdominal pain |  |  | - |
| Grade 1-2 | 2 | 2 |  |
| Grade 3-4 | 0 | 0 |  |
| Vomiting |  |  | - |
| Grade 1-2 | 4 | 1 |  |
| Grade 3-4 | 0 | 0 |  |
| Bowel obstruction |  |  | - |
| Grade 1-2 | 1 | 0 |  |
| Grade 3-4 | 1 | 2 |  |
| Diarrhea |  |  | - |
| Grade 1-2 | 1 | 1 |  |
| Grade 3-4 | 0 | 0 |  |
| Increased aminotransferase |  |  | 0.557 |
| Grade 1-2 | 11 | 4 |  |
| Grade 3-4 | 2 | 2 |  |
| **Endocrinal** |  |  |  |
| Hypothyroidism |  |  | - |
| Grade 1-2 | 4 | 3 |  |
| Grade 3-4 | 0 | 0 |  |
| Hyperthyroidism |  |  | - |
| Grade 1-2 | 0 | 0 |  |
| Grade 3-4 | 0 | 0 |  |
| Hypercortisolism |  |  | - |
| Grade 1-2 | 1 | 1 |  |
| Grade 3-4 | 0 | 0 |  |
| Hyperglycaemia |  |  | - |
| Grade 1-2 | 0 | 1 |  |
| Grade 3-4 | 0 | 0 |  |
| Adrenal insufficiency |  |  | - |
| Grade 1-2 | 2 | 2 |  |
| Grade 3-4 | 0 | 0 |  |
| **Respiratory** |  |  |  |
| Cough |  |  | - |
| Grade 1-2 | 2 | 1 |  |
| Grade 3-4 | 0 | 0 |  |
| Pneumonia |  |  | - |
| Grade 1-2 | 1 | 2 |  |
| Grade 3-4 | 0 | 0 |  |
| **Others** |  |  |  |
| Leucopenia |  |  | - |
| Grade 1-2 | 2 | 4 |  |
| Grade 3-4 | 0 | 0 |  |
| Hypertension |  |  | - |
| Grade 1-2 | 3 | 4 |  |
| Grade 3-4 | 0 | 0 |  |
| Renal Insufficiency |  |  | - |
| Grade 1-2 | 0 | 1 |  |
| Grade 3-4 | 0 | 0 |  |
| Elevated lipase |  |  | - |
| Grade 1-2 | 0 | 1 |  |
| Grade 3-4 | 0 | 0 |  |
